# Case of Phrenitis from Injury of the Head

**Published:** 1814-05

**Authors:** 


*For the Medical and Physical Journal.*

### *Case of Phrenitis from Injury of the Head.*

"Nullum vulnus capitis contemnendum."

ON a Monday in the month of August, William Slater, a boy in the employment of an apothecary of this town recently deceased, whilst in the act of putting up his master's shutters, fell into the area. He was stunned by the fall, and lost a considerable quantity of bloody fluid from the right ear. When sufficiently recovered to be able to walk, he was led home (a short distance) by the servant of the house. He had no recollection of the mode in which the accident happened. From this day until the following Wednesday he kept his bed, complaining of violent pain, and particularly of a burning sensation in the upper part of the head, with constant vomiting. On Thursday was persuaded to walk out a little, which he did for about half an hour. On Friday he became worse, and from this time, till the afternoon of Monday, he continued nearly in the same state.

During the whole of this period he was seen at intervals by his master, who thought so lightly of the case that he attempted nothing for his relief. On Monday evening he became furiously delirious, though at times he was sensible; and in answer to questions put to him, complained of acute pain in the head. On Tuesday I saw him. He was then raving. His pulse was weak, quick, and undulating; the tongue white and tremulous; the countenance had the appearance of one sinking in the last stage of typhus; at times he screamed violently, at others uttered more distinct exclamations—O my head! When roused he knew his mother, and being desired to put out his tongue and move his limbs, did both, but soon relapsed into his former condition. Nothing could be retained on the stomach.

With symptoms so evidently characteristic of an advanced stage of inflamed brain, no reasonable expectation of benefit could be entertained. The weakness of the pulse seemed at first to forbid the use of bleeding; and admitting the expediency of having recourse to it, its utility was very questionable at this late period of the disease.*

Twelve leeches were applied to the temples, the head was shaved, and a blister applied, previous to which, on exa-

* I have since had many cases of internal inflammation under my care, which have proved this opinion to be incorrect. From these I am led to the conclusion that, generally speaking, the state of the pulse should never deter us in the employment of bleeding, where evident marks of inflammatory action are present.

mining mining the parts about the ear of the right side, a fissure *without* depression was discovered. On Wednesday morning was somewhat more tranquil. Sixteen ounces of blood were taken from the arm, but the evening brought with it a return of all the symptoms. His fæces and urine were passed involuntarily; his tongue was parched and black; and he had slight convulsive motions of the whole body. On Saturday he died.

On dissection, the membranes of the brain were found remarkably inflamed, with depositions of coagulable lymph between the pia mater and arachnoid membrane. The appearance of the membrane, with this deposition, very much resembled that of a portion of the heart with fat on its surface. A part having been removed, it was not recognised by some medical friends to whom it was shewn. Water was effused into the ventricles. The fissure extended into the base of the brain; we found a coagulum. Blood was effused at the part where the blow was apparently received, near the inferior angle of the parietal bone.

How far this boy's life might have been saved by the employment of bleeding immediately after the accident, and by its repetition according to the violence of the symptoms, is a question which it is impossible to determine. It is obvious, however, that the neglect of it, in a case so strongly marked from its commencement, is a proof of gross ignorance or extreme carelessness on the part of the practitioner; but "de mortuis nil nisi bonum." J. W.

*London, March* 10, 1814.

